# *Mycobacterium tuberculosis* Responds to Chloride and pH as Synergistic Cues to the Immune Status of its Host Cell

**DOI:** 10.1371/journal.ppat.1003282

**Published:** 2013-04-04

**Authors:** Shumin Tan, Neelima Sukumar, Robert B. Abramovitch, Tanya Parish, David G. Russell

**Affiliations:** 1 Cornell University, College of Veterinary Medicine, Department of Microbiology and Immunology, Ithaca, New York, United States of America; 2 Infectious Disease Research Institute, and Department of Global Health, University of Washington School of Medicine, Seattle, Washington United States of America; Johns Hopkins School of Medicine, United States of America

## Abstract

The ability of *Mycobacterium tuberculosis* (Mtb) to thrive in its phagosomal niche is critical for its establishment of a chronic infection. This requires that Mtb senses and responds to intraphagosomal signals such as pH. We hypothesized that Mtb would respond to additional intraphagosomal factors that correlate with maturation. Here, we demonstrate that [Cl^−^] and pH correlate inversely with phagosome maturation, and identify Cl^−^ as a novel environmental cue for Mtb. Mtb responds to Cl^−^ and pH synergistically, in part through the activity of the two-component regulator *phoPR*. Following identification of promoters responsive to Cl^−^ and pH, we generated a reporter Mtb strain that detected immune-mediated changes in the phagosomal environment during infection in a mouse model. Our study establishes Cl^−^ and pH as linked environmental cues for Mtb, and illustrates the utility of reporter bacterial strains for the study of Mtb-host interactions *in vivo*.

## Author Summary

*Mycobacterium tuberculosis* (Mtb) is the causative agent of tuberculosis, a disease that remains a major global health problem. To ensure its long-term survival in the host, Mtb must be able to sense and respond to changes in its immediate environment, such as the pH differences that occur in the phagosome in which it lives. Knowledge of the external signals that Mtb recognizes during infection is critical for understanding the impact of the microenvironment on Mtb pathogenesis and persistence, and how Mtb interacts with its host cell. We show here that [Cl^−^] correlates inversely with pH as the phagosome matures, and identify [Cl^−^] as a novel cue that Mtb responds to, in synergism with pH. By constructing a Mtb strain that fluorescently reports on changes in [Cl^−^] and pH, we find using a mouse model of infection that environmental alterations in Mtb's phagosomal home are mediated at the local level by activities of the host immune system. Our study demonstrates how a pathogen can exploit linked environmental cues during infection, and shows the value of reporter bacterial strains for Mtb-host whole animal studies.

## Introduction

*Mycobacterium tuberculosis* (Mtb) causes a chronic infection in approximately one third of the human population and remains an important public health problem [1]. The macrophage (MØ) is the major host cell for much of Mtb's life cycle, and a defining feature of Mtb's pathogenesis is its ability to arrest full maturation of the phagosome in which it resides [2], [3]. Indeed, Mtb mutants that fail to arrest phagosomal maturation have reduced survival during MØ infection [4]. However, Mtb remains subject to multiple stresses within the phagosome, which may act as important environmental cues for Mtb [5]. Proper sensing of such signals informs Mtb of its surroundings, allowing the bacterium to respond appropriately to ensure its survival and replication. Elucidating the cues that Mtb recognizes during infection, and the possible interplay between such signals, is critical for a complete understanding of the impact of the microenvironment on Mtb pathogenesis and persistence, and Mtb's interaction with fundamental host cell processes.

One environmental cue that has received particular attention is pH; the Mtb phagosome acidifies to an intermediate pH of 6.4 [3], [4], [6], and even in medium, the bacterium exhibits a profound transcriptional response to acidic pH [5], [7], [8]. The abolition of phagosome acidification during bacterial uptake by MØs, through treatment with concanamycin A, eliminates a majority of Mtb's transcriptional response, indicating the importance of pH as a signal for the bacterium in sensing and responding to its environment [5]. The process of acidification does not, however, proceed in isolation. Specifically, acidification (increase in [H^+^]) must be counterbalanced by efflux of other cations from the phagosome, and/or by the uptake of a counter anion. We hypothesized that Mtb might also take advantage of this counterbalancing factor as an environmental cue, expanding the sensitivity and dynamic range of its ability to define its immediate environment. Cell biological studies have established Cl^−^ as a major counter anion during acidification of the endosome [9]–[11]. Several Cl^−^ channels are known to be present on the endosomal membrane [12], [13], although it remains controversial which of these channels are involved in the counter-balancing of increased [H^+^] during endosomal maturation [14], [15]. More recent studies have also proposed efflux of cations, such as K^+^, as a counter mechanism to increased [H^+^] in the lysosome [16]. The existence of such mechanisms have not, however, been formally shown for phagosomes. In this context, it is of particular note that the Mtb phagosome has been reported to possess a high [Cl^−^] [17]. The impact of common ions and changes in their concentration on Mtb during infection is a concept that is just beginning to be appreciated [18]; however, much remains to be determined regarding their physiological significance.

In this study, we show that [Cl^−^] increases during phagosome maturation, mirroring a decrease in pH within the compartment. Mtb modulates its transcriptional profile in response to [Cl^−^], and reacts to the environmental cues of pH and [Cl^−^] in a synergistic manner, with the two-component regulatory system *phoPR* playing a central role in this response. By constructing a fluorescent reporter Mtb strain responsive to both Cl^−^ and pH, we were further able to directly examine the microenvironment of Mtb during *in vivo* infection in a mouse model. Maturation of Mtb-containing phagosomes is known to be impacted by the immune status of the MØ. Infection of wild type versus immune-deficient interferon-γ^−/−^ mice revealed differential induction of fluorescence *in vivo*, and demonstrated the influence of host immune pressure on the microenvironment in which Mtb resides. These data were further validated with a second Mtb reporter strain, expressing GFP under the regulation of the more fully-characterized hypoxia and nitric oxide-responsive *dosR* regulon [19]–[22]. The results confirm existing hypotheses concerning localized immune-mediated pressure within infection foci, and provide a new generation of tools to probe the fitness and viability of Mtb in *in vivo* infection models.

## Results/Discussion

### [Cl^−^] increases during phagosomal maturation

**Figure 1:**
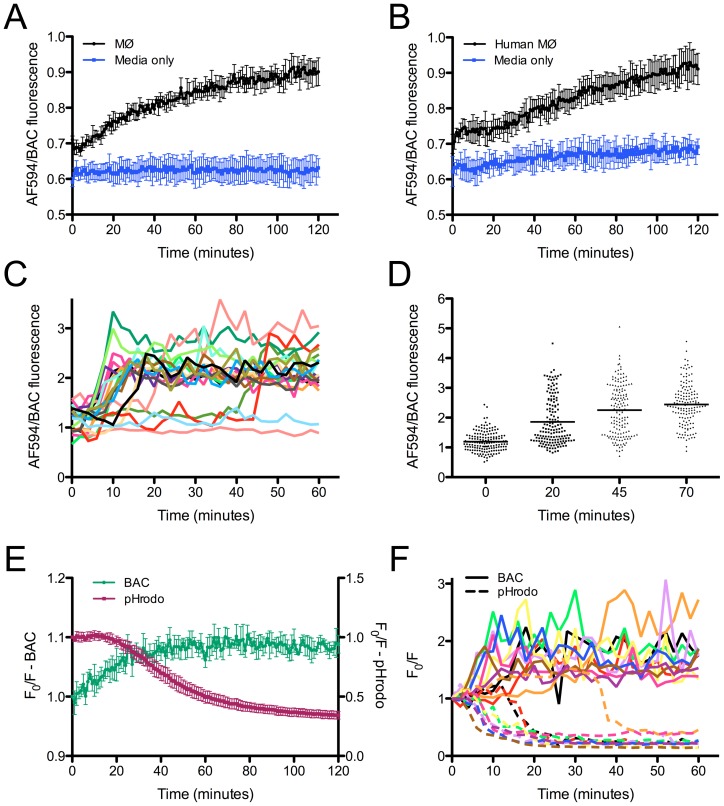
[Cl^−^] increases and pH decreases during phagosome maturation. (A and B) [Cl^−^] increases during phagosome maturation. BAC/AF594 beads were added to murine bone marrow-derived MØs (A) or MØs derived from human monocytes (B). In each case, sensor beads were also added to wells containing only media, with no MØs (“Media only”). BAC (Cl^−^-sensitive) and AF594 (calibration fluorophore) fluorescence were tracked with a microplate reader over time. Data are shown as means ± SD from 4 wells. (C) Single Cl^−^ sensor bead tracking during phagosome maturation. Cl^−^ sensor beads were added to murine bone marrow-derived MØs and fluorescence for individual beads tracked over time by live-cell time-lapse microscopy (see Video S1). Each line on the graph represents a single bead. (D) Heterogeneity of [Cl^−^] in individual phagosomes. BAC/AF594 beads were added to murine bone marrow-derived MØs and fluorescence measured at indicated time points. Each point on the graph represents a single bead. Bars represent mean values. (E) [Cl^−^] and pH are inversely correlated during phagosome maturation. BAC/pHrodo beads were added to murine bone marrow-derived MØs and BAC (green) and pHrodo (red) fluorescence tracked with a microplate reader over time. F_0_ is fluorescence at time = 0 min, and F is fluorescence at each given time point. Data are shown as means ± SD from 4 wells. (F) Single BAC/pHrodo bead tracking during phagosome maturation. BAC/pHrodo beads were added to murine bone marrow-derived MØs and fluorescence for individual beads tracked over time by live-cell time-lapse microscopy (see Video S3). F_0_ is fluorescence at time = 0 min, and F is fluorescence at each given time point. Each color represents a single bead, with solid lines tracking BAC signal and dashed lines tracking pHrodo signal from the same bead.

We first sought to establish the dynamics of [Cl^−^] during maturation of the phagosome with model particles. The fluorescent Cl^−^-sensitive, pH-insensitive compound 10,10′-Bis[3-carboxylpropyl]-9,9′-biacridinium (BAC) [9] was synthesized as a trifluoroacetate salt, and coupled to IgG beads. As previously reported, BAC fluorescence is quenched by Cl^−^ in a concentration-dependent manner, and is unaffected by pH changes (Figure S1 in Text S1) [9]. To track [Cl^−^] changes during phagosomal maturation we attached Alexa Fluor 594 (AF594) as a calibration fluorophore to the BAC-IgG beads. These dual-color Cl^−^ sensor beads were added to murine bone marrow-derived MØs and fluorescence measured in a microplate reader. We observed an increase in AF594/BAC fluorescence ratios over time, indicating an increase in [Cl^−^] as the phagosome matured (Figure 1A). This increase in [Cl^−^] was also observed with phagosome maturation in MØs derived from human monocytes (Figure 1B). To calibrate AF594/BAC ratios to actual [Cl^−^], we treated MØs that had phagocytosed Cl^−^ sensor beads with bafilomycin A1 and the ionophores nigericin and monensin in buffers of known [Cl^−^]. By fitting a polynomial regression to the standard curve (Figure S2 in Text S1), we calculate that phagosomal [Cl^−^] reached a maximal concentration of ∼70–95 mM. As this is a population-based measurement, we note that this value range underestimates the [Cl^−^] that can be reached in individual phagosomes (see below).

We further examined the dynamics of [Cl^−^] increase during phagosome maturation by tracking individual beads during phagocytosis by live-cell time-lapse microscopy. These experiments showed that the switch from low [Cl^−^] to high [Cl^−^] occurred for most beads, although a subset remained in phagosomes with low [Cl^−^] (Figure 1C and Video S1). Imaging of populations of Cl^−^ sensor bead-containing cells at given time points illustrated the heterogeneity in [Cl^−^] attained in individual phagosomes, with measurements indicating that a [Cl^−^] greater than 120 mM was reached in some phagosomes (Figure 1D). Similar results were obtained in MØs derived from human monocytes (data not shown). Cl^−^ sensor beads present in media alone and imaged in parallel did not show significant changes in fluorescence, demonstrating that the decrease in BAC fluorescence observed in the phagocytosed beads has a biological basis and is not due to bleaching of the fluorescent signal during imaging (Video S2).

In examining these results, we noted that the increase in [Cl^−^] mirrored the kinetics of the decrease in phagosomal pH previously reported [23]. In order to quantify this correlation directly within a single experiment, we coupled BAC to IgG beads in combination with the red fluorescent pH sensor pHrodo, which exhibits an increase in fluorescence as pH decreases. Measurement of the fluorescence profile of the beads during phagocytosis by MØs showed the previously observed quenching of BAC signal indicative of increased [Cl^−^] as the phagosome matured (Figure 1E). pHrodo fluorescence on the same particles exhibited an inverse profile, increasing in intensity over time, signaling a decrease in pH (Figure 1E). Analysis of the phagocytosis of the BAC/pHrodo indicator beads by live-cell time-lapse microscopy further verified these results at the individual phagosome level (Figure 1F and Video S3). Similar profiles were observed in MØs derived from human monocytes (data not shown). We also verified that BAC/pHrodo beads imaged in media alone did not show such changes in fluorescence profile (Video S4).

Further support for the relation between [Cl^−^] and pH during phagosomal maturation was demonstrated by the failure of phagosomal [Cl^−^] to increase when MØs were treated with bafilomycin A1 (Figure S3A in Text S1). Similarly, addition of bafilomycin A1 to the MØs after phagosomes containing the Cl^−^ sensor beads had initially been allowed to mature resulted in increased BAC fluorescence, indicating a reversal of the Cl^−^ accumulation upon dissipation of the pH gradient (Figure S3B in Text S1). Together, these results demonstrate that [Cl^−^] increases during phagosomal maturation, and supports the proposed functional relationship between acidification of the endosomes and [Cl^−^] increase [9]–[11].

### Mtb regulates gene expression in response to Cl^−^, with a subset corresponding to pH responsive genes

Mtb shows a marked transcriptional response upon exposure to acidic pH, and we have previously shown that almost half of the Mtb genes upregulated during an early stage of MØ infection are induced in a pH-dependent manner [5]. Given our results indicating [Cl^−^] increase during phagosomal maturation and the link between [Cl^−^] and acidification, we compared the transcriptional profiles of Mtb grown in regular 7H9 media to those grown in 7H9 media supplemented with 250 mM NaCl for 4 hours. The number of genes (32) upregulated on exposure to high [Cl^−^] was noticeably fewer than the hundreds previously reported to be induced under acidic pH (Table 1) [5], [7]. Strikingly however, a significant number of genes that were upregulated in the presence of high [Cl^−^] (18/32) were genes that also showed increased expression during exposure to acidic pH (Table 1).

**Table 1:** Overlap between Mtb genes upregulated on exposure to high [Cl^−^] or acidic pH.

Gene Name	Ratio	Acidic pH induced	Description
*MT0772.5*	1.59	+	PE-PGRS family protein
*MT1178*	1.36	−	HP
*MT1746.1*	1.73	−	HP
*MT2423.1*	1.34	−	HP
*MT3106.1*	1.44	+	PE family protein
*Rv0263c*	1.42	+	CHP
*Rv0264c*	1.47	+	CHP
*Rv0516c*	1.58	−	CHP
*Rv1057*	1.61	+	CHP
*Rv1115*	1.51	−	HP
*Rv1187* (*rocA*)	1.37	+	Pyrroline-5-carboxylate dehydrogenase
*Rv1376*	1.50	−	CHP
*Rv1403c*	1.96	+	Methyltransferase
*Rv1405c*	2.98	+	Methyltransferase
*Rv1497* (*lipL*)	1.36	−	esterase
*Rv1577*	1.30	+	phiRv1 phage protein
*Rv1705c* (*ppe22*)	1.86	−	PPE family protein
*Rv1706c* (*ppe23*)	1.33	+	PPE family protein
*Rv2389c* (*rpfD*)	1.31	+	Resuscitation promoting factor
*Rv2390c*	2.00	+	CHP
*Rv2450c* (*rpfE*)	1.31	−	Resuscitation promoting factor
*Rv2549c*	1.35	−	CHP
*Rv2651c*	1.60	−	phiRv2 phage protease
*Rv3093c*	1.44	−	Oxidoreductase
*Rv3252c* (*alkB*)	1.33	+	Transmembrane alkane 1-monooxygenase
*Rv3429* (*ppe59*)	1.33	−	PPE family protein
*Rv3613c*	1.48	+	HP
*Rv3614c*	1.53	+	CHP
*Rv3615c*	1.51	+	CHP
*Rv3616c*	1.49	+	CHP
*Rv3746c* (*pe34*)	1.92	+	PE family protein
*Rv3841* (*bfrB*)	1.38	−	bacterioferritin

List of genes upregulated >1.3 fold on exposure to 250 mM NaCl for 4 hrs (p<0.05). Genes induced (+) or unchanged (−) by acidic pH, as determined by comparison to references [5], [7]. HP, hypothetical protein. CHP, conserved hypothetical protein.

The upregulated gene expression detected by microarrays was validated by semi-quantitative real time PCR (qRT-PCR) for several genes. These experiments were also carried out on samples exposed to acidic pH (pH 5.7), and showed data consistent with the microarray analysis (Figure S4 in Text S1). While our microarray platform allows for the global analyses of gene expression changes, it does have a flattened dynamic range [5], [24], and the qRT-PCR data indicate that the actual level of induction is considerably greater. These experiments indicate that Mtb responds transcriptionally to Cl^−^, and further reinforce the idea that pH and Cl^−^ may function as interconnected environmental cues for Mtb during the course of infection.

### Mtb carrying an *rv2390c* promoter-GFP fusion functions as a Cl^−^ and pH reporter

**Figure 2:**
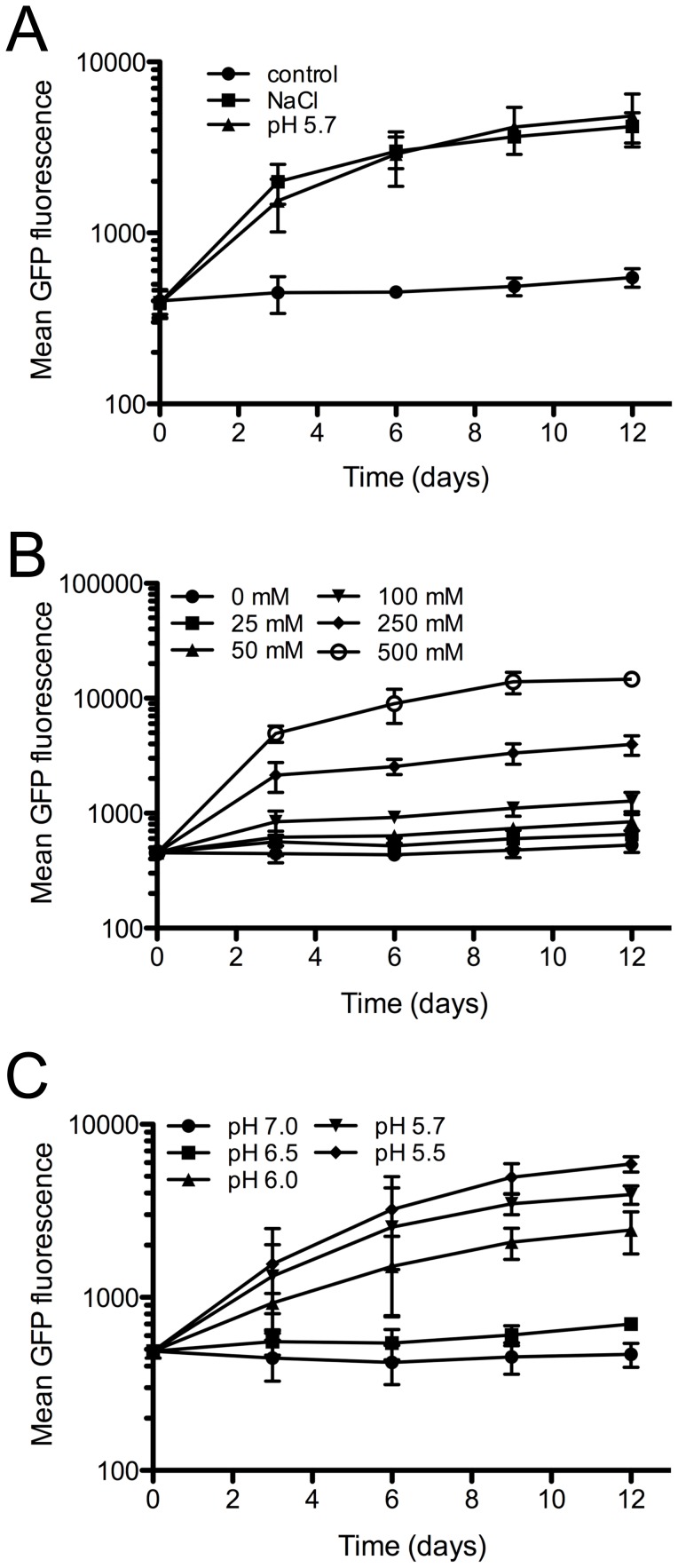
An *rv2390c*'::GFP strain functions as a Cl^−^ and pH responsive reporter Mtb. (A) Mtb responds to Cl^−^ and pH. CDC1551(*rv2390c*'::GFP) was grown *in vitro* in media at pH 7.0 (control, circles), pH 7.0+250 mM NaCl (squares), or pH 5.7 (triangles). Samples were taken over time, fixed, and GFP signal analyzed by FACS. Data are shown as means ± SD from 3 independent experiments. (B and C) Mtb responds to Cl^−^ and pH in a concentration-dependent manner. CDC1551(*rv2390c*'::GFP) was grown *in vitro* in media at pH 7.0 supplemented with different [NaCl] (B), or in media at different pH (C). Samples were analyzed as in (A). Data are shown as means ± SD from 3 independent experiments.

To perform analyses of Cl^−^ and pH as environmental cues for live Mtb, we utilized the microarray and qRT-PCR results to select candidate genes for construction of a reporter Mtb strain that would be responsive to both changes in [Cl^−^] and pH. We focused on the *rv2390c-rpfD* operon, which appeared particular promising as both genes in the operon showed robust induction under conditions of high [Cl^−^] or acidic pH (Figure S4 in Text S1). The promoter region of *rv2390c* was cloned upstream of GFP in a replicating plasmid, and transformed into Mtb CDC1551. This CDC1551(*rv2390c*'::GFP) reporter strain was then grown in media +/− 250 mM NaCl, buffered to pH 7.0 to study [Cl^−^] effects at neutral pH, or in media buffered at pH 5.7, without added NaCl. Using FACS analysis, we observed an increase in GFP fluorescence of CDC1551(*rv2390c*'::GFP) in conditions of high [Cl^−^] or acidic pH over time, with peak inductions of 7–9 fold over control in each instance (Figure 2A). To verify the Cl^−^-specificity of the response, we tested several other compounds for their ability to induce GFP fluorescence in CDC1551(*rv2390c*'::GFP), including KCl, arginine-HCl, Na_2_SO_4_, and sucrose, in media buffered at pH 7.0. Induction was observed with compounds containing Cl^−^, but not with Na_2_SO_4_ and sucrose, indicating that Cl^−^ was the agent responsible for the increase in GFP signal, and suggesting that neither Na^+^ nor osmolarity were contributory factors (Figure S5 in Text S1). Induction of *rv2390c*'::GFP expression was also reversible, with GFP fluorescence returning to baseline levels within 5 days of removal of the high [Cl^−^] stimulus in log-phase bacteria (Figure S6 in Text S1). These data, along with the lack of induction observed with other stressors such as NO and hypoxia (Figure S7 in Text S1), argue for the usefulness of CDC1551(*rv2390c*'::GFP) as a specific reporter Mtb strain for the intraphagosomal cues of pH and Cl^−^.

To determine if Mtb's response to Cl^−^ occurs in a concentration-dependent manner, we repeated the time-course induction assays with media containing different [Cl^−^] at pH 7.0. GFP fluorescence of CDC1551(*rv2390c*'::GFP) increased as [Cl^−^] rose, showing Mtb's ability to modulate its response to [Cl^−^] in a manner comparable to a rheostat (Figure 2B). In agreement with a previous study reporting Mtb's dynamic response to diminishing pH [24], we also observed increasing GFP signal with decreasing pH for CDC1551(*rv2390c*'::GFP) (Figure 2C). These results further demonstrate the usefulness of CDC1551(*rv2390c*'::GFP) as a reporter Mtb strain for Cl^−^ and pH, and indicate that Mtb's response to these two environmental cues is fine-tuned by its environment.

### Cl^−^ and pH act synergistically as environmental cues for Mtb

**Figure 3:**
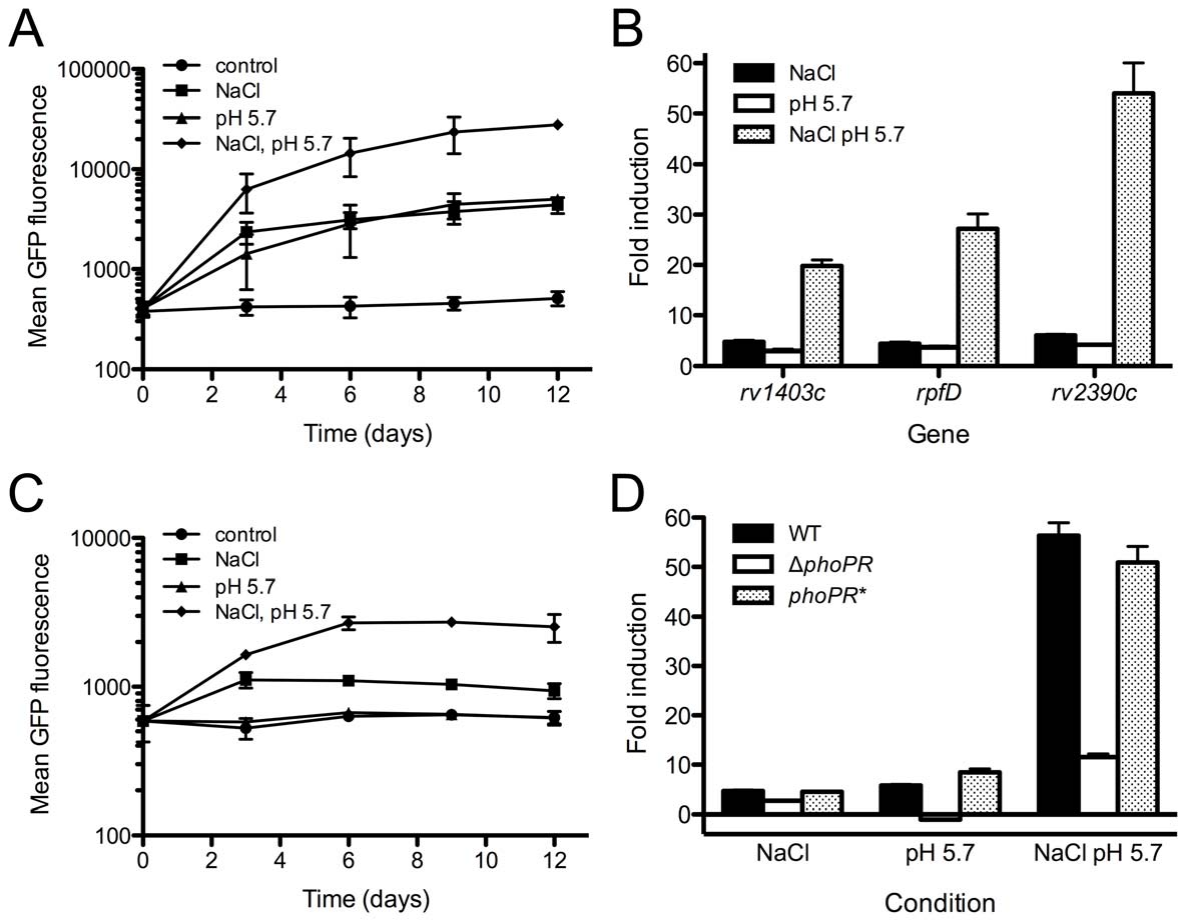
Links between Mtb's response to Cl^−^ and pH. (A) Mtb responds synergistically to Cl^−^ and pH. CDC1551(*rv2390c*'::GFP) was grown *in vitro* in media at pH 7.0 (control, circles), pH 7.0+250 mM NaCl (squares), pH 5.7 (triangles), or pH 5.7+250 mM NaCl (diamonds). Samples were taken over time, fixed, and GFP signal analyzed by FACS. Data are shown as means ± SD from 3 independent experiments. (B) Mtb's synergistic response to Cl^−^ and pH is reflected transcriptionally. qRT-PCR of gene expression in WT grown as in (A) for 4 hrs. Fold induction is as compared to WT grown in media at pH 7.0. Data are shown as means ± SD from 3 technical replicates. (C) *phoPR* is required for Mtb's response to pH and plays a role in its Cl^−^ response. CDC1551(*rv2390c*'::GFP, *phoP*::Tn) was grown *in vitro* in media at pH 7.0 (control, circles), pH 7.0+250 mM NaCl (squares), pH 5.7 (triangles), or pH 5.7+250 mM NaCl (diamonds). Samples were taken over time, fixed, and GFP signal analyzed by FACS. Data are shown as means ± SD from 3 independent experiments. (D) Complementation of Δ*phoPR* restores Mtb's response to pH and Cl^−^. qRT-PCR of *rv2390c* expression in WT, Δ*phoPR*, and the complemented mutant (phoPR*) grown as in (C) for 4 hrs. Fold induction is as compared to the corresponding strain grown in media at pH 7.0. Data are shown as means ± SD from 3 technical replicates.

To test whether Cl^−^ and pH might act synergistically as intraphagosomal cues, we incubated CDC1551(*rv2390c*'::GFP) in media buffered at pH 5.7, with 250 mM NaCl. These conditions resulted in induction of GFP fluorescence to a level (>50 fold) much greater than merely the sum of the GFP signal obtained when the bacteria were grown in conditions with only one cue (high [Cl^−^] or acidic pH) (Figure 3A). qRT-PCR tests on several genes in wild type Mtb (WT) exposed to the different conditions confirmed the synergistic activity (Figure 3B).

This synergy implied cross-talk between regulatory circuits. In particular, we examined the role of the two-component regulator *phoPR*, a system previously shown to be required for expression of the acid and phagosome-regulated locus *aprABC* [24], and whose regulon significantly overlaps the list of genes regulated in a pH-dependent manner during MØ infection [5], [8]. We found that unlike WT, a *phoP*::Tn mutant carrying the *rv2390c*'::GFP reporter failed to induce GFP fluorescence during growth at acidic pH, supporting the critical role of *phoP* in regulating Mtb's response to pH (Figure 3C). Our experiments further indicated that *phoP* also played a role in regulating Mtb's response to Cl^−^, as induction of the GFP reporter signal during growth in high [Cl^−^] was reduced in the *phoP*::Tn mutant as compared to WT (1.5–2 fold vs. 7–9 fold) (Figure 3C). Intriguingly, GFP induction with the reporter *phoP*::Tn mutant in conditions of high [Cl^−^] at acidic pH (4 fold) was still greater than that observed with high [Cl^−^] alone, despite the lack of induction with acidic pH as a single signal (Figure 3C). qRT-PCR analyses on a Δ*phoPR* Mtb mutant, as well as a complemented Δ*phoPR* strain (phoPR*), confirmed these data. There was decreased induction of target transcript in conditions of high [Cl^−^] alone or high [Cl^−^] at acidic pH in the Δ*phoPR* mutant as compared to WT (3 vs. 5 fold and 12 vs. >50 fold respectively), and no increase in transcript at acidic pH for the mutant (Figure 3D). Genetic complementation restored transcript induction in the mutant to WT levels (Figure 3D).

These results implicate *phoPR* as a regulator that modulates Mtb's response to Cl^−^, while also indicating that it is merely one part of a regulatory circuit that impacts this response.

### [Cl^−^] and pH in the Mtb phagosome change during MØ infection

Having established that Mtb's response to Cl^−^ and pH is interconnected *in vitro*, we next pursued these studies in the context of MØ infection by Mtb. To make use of the *rv2390c*'::GFP reporter for these intracellular studies, we first moved the construct into a replicating plasmid containing mCherry driven by the constitutive promoter *smyc* [24], [25], to generate the strain CDC1551(*rv2390c*'::GFP, *smyc*'::mCherry) (Figure S8 in Text S1). This allows visualization of all bacteria regardless of reporter induction levels, and an internal calibration of the GFP signal.

**Figure 4:**
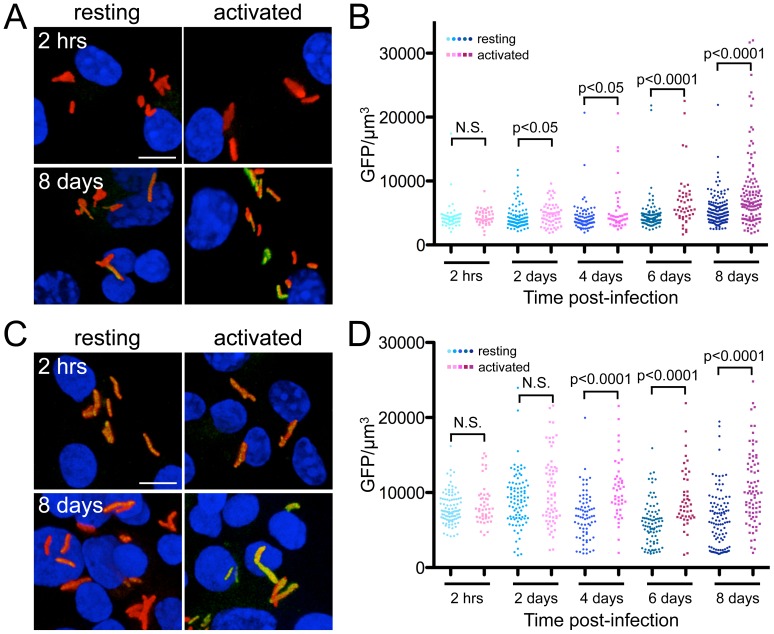
[Cl^−^] and pH in the Mtb phagosome change during MØ infection. (A and B) Expression of *rv2390c-rpfD* is upregulated during Mtb infection of activated vs. resting MØs. Resting or activated murine MØs were infected with CDC1551(*rv2390c*'::GFP, *smyc*'::mCherry). (A) shows 3D confocal images of the infection at the beginning (2 hrs) and end (8 days) of the infection. All bacteria are marked in red (*smyc*'::mCherry), the reporter is shown in green (*rv2390c*'::GFP), and nuclei are shown in blue (DAPI). Scale bar 5 µm. (B) shows quantification of the GFP/µm^3^ signal for each bacterium measured from multiple 3D confocal images. Each point on the graph represents a bacterium or a tightly clustered group of bacteria (circles – Mtb in resting MØs, squares – Mtb in activated MØs). p-values were obtained with a Mann-Whitney statistical test. (C and D) Mtb pre-treatment with high [Cl^−^] leads to divergent *rv2390c*'::GFP signal during infection of resting vs. activated MØs. CDC1551(*rv2390c*'::GFP, *smyc*'::mCherry) was exposed to 250 mM NaCl for 6 days prior to infecting resting or activated murine MØs. (C) shows 3D confocal images of the infection at the beginning (2 hrs) and end (8 days) of the infection. All bacteria are marked in red (*smyc*'::mCherry), the reporter is shown in green (*rv2390c*'::GFP), and nuclei are shown in blue (DAPI). Scale bar 5 µm. (D) shows quantification of the bacterial GFP/µm^3^ signal, determined as in (B). p-values were obtained with a Mann-Whitney statistical test.

Activation of MØs prior to infection with Mtb is known to increase the maturation stage and lower the pH of the bacteria-containing vacuoles [26], [27], which should increase induction of GFP expression as a function of both pH and [Cl^−^]. Resting or activated murine bone marrow-derived MØs were infected with the reporter Mtb strain, and samples examined by confocal microscopy. We observed increased GFP fluorescence as the infection progressed, with significantly more induction of GFP signal in the activated MØs (Figures 4A and 4B). This difference in the microenvironment experienced by Mtb during infection of resting or activated MØ was even more starkly illustrated by pre-incubating the reporter Mtb in conditions of high [Cl^−^] prior to MØ infection. In this case, the inoculating bacteria had an increased level of *rv2390c*'-driven GFP expression at the start of infection, and exhibited an enhanced divergence in GFP signal between the resting and activated MØs (Figures 4C and 4D).

These experiments indicate that Mtb experiences different [Cl^−^] and pH during MØ infection, dependent on the activation status of the host MØ, and points to dynamic regulation of its gene expression in response to these environmental cues.

### Reporter Mtb strains directly reveal Mtb's microenvironment during *in vivo* infection, and demonstrate the impact of host immune pressure on environmental cues

The MØ experiments above demonstrate the feasibility of using the CDC1551(*rv2390c*'::GFP, *smyc*'::mCherry) reporter strain to reveal important aspects of Mtb's microenvironment during infection. We sought to test the utility of this reporter system in a whole animal infection where the infection foci will likely present regional variation in immune responsiveness and heterogeneous levels of MØ activation. To probe if we could detect regional variation in immune-mediated modulation of infected MØs, we infected C57BL/6J WT or isogenic interferon-γ^−/−^ (IFNγ^−/−^) mice with Erdman(*rv2390c*'::GFP, *smyc*'::mCherry) via intranasal inoculation. IFNγ^−/−^ mice fail to properly activate their MØs on infection and are susceptible to Mtb, developing a disseminated infection that is fatal [28], [29]. The Erdman strain was used for these experiments, as it establishes robust infection in mice. *In vitro* tests show that the Erdman reporter strain responds similarly to both Cl^−^ and pH (Figure S9 in Text S1).

**Figure 5:**
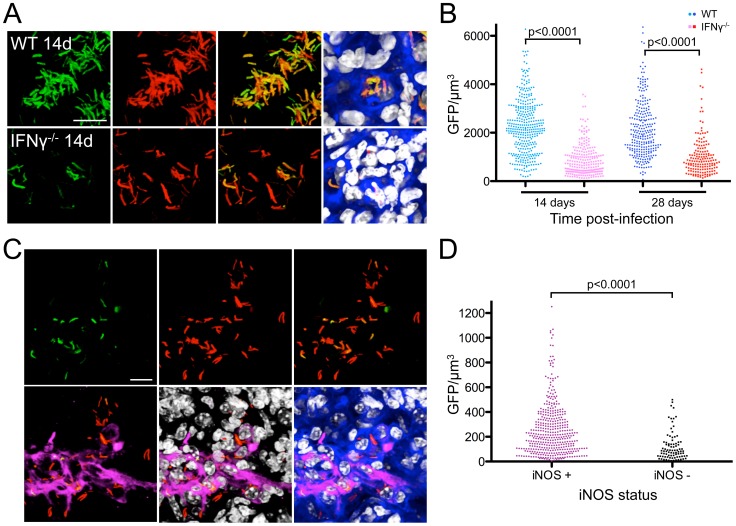
Reporter Mtb strains illustrate the link between immune pressure and bacterial microenvironment. (A and B) Greater induction of *rv2390c*'::GFP during Mtb infection of WT vs. IFNγ^−/−^ mice. WT or IFNγ^−/−^ C57BL/6J mice were infected with Erdman(*rv2390c*'::GFP, *smyc*'::mCherry) for up to 28 days. (A) shows 3D confocal images from a 14 day infection. All bacteria are marked in red (*smyc*'::mCherry), the reporter is shown in green (*rv2390c*'::GFP), nuclei are shown in grayscale (DAPI), and phalloidin staining of f-actin is shown in blue. Scale bar 10 µm. (B) shows quantification of the GFP/µm^3^ signal for each bacterium measured from multiple 3D confocal images, at 14 or 28 days post-infection. Each point on the graph represents a bacterium or a tightly clustered group of bacteria (circles – Mtb in WT mice, squares – Mtb in IFNγ^−/−^ mice). p-values were obtained with a Mann-Whitney statistical test. (C and D) Immune activation upregulates *rv2390c*'::GFP induction. C57BL6/J WT mice were infected with Erdman(*rv2390c*'::GFP, *smyc*'::mCherry) for 28 days. (C) shows 3D confocal images of the infection with bacteria marked in red (*smyc*'::mCherry), reporter signal shown in green (*rv2390c*'::GFP), iNOS stained in magenta, nuclei shown in grayscale (DAPI), and phalloidin staining of f-actin shown in blue. Scale bar 10 µm. (D) shows quantification of the bacterial GFP/µm^3^, determined as in (B), in Mtb present in iNOS-positive vs. negative regions. p-values were obtained with a Mann-Whitney statistical test.

Infected mice were sacrificed at 14 and 28 days post-challenge, and lung tissue examined by confocal microscopy. We observed significantly higher GFP fluorescence in the reporter strain in WT vs. IFNγ^−/−^ mice at each time point examined (Figures 5A and 5B). In the case of IFNγ^−/−^ mice, we also noted a disseminated infection, in agreement with previous studies (Figure 5A) [28], [29]. These results faithfully reproduce our MØ experiments since IFNγ^−/−^ mice, which are unable to activate their MØs, exhibit reduced expression of the GFP reporter signal.

To further examine the impact of host immune pressure on determining Mtb's microenvironment, we used host inducible nitric oxide synthase (iNOS) expression as an indicator of immune activation in WT mice at 28 days post-infection. This allowed us to compare Mtb resident in regions with vs. without an active immune response, within a single infected WT host. A first observation was that most Mtb were located in iNOS-positive regions in the mouse lung tissue (Figures 5C and 5D). Significantly however, we found greater reporter GFP fluorescence in the bacteria residing in iNOS-positive regions vs. those located in iNOS-negative regions (Figure 5D). This result reinforces the concept that host immune pressure can impact substantially on the cues that Mtb responds to in its microenvironment, and that reporter Mtb strains can be exploited to shed light on the signals the bacteria are exposed to during *in vivo* infection. In particular in the context of the *rv2390c*'::GFP reporter, it suggests that Mtb experiences a microenvironment with higher [Cl^−^] and more acidic pH during infection of a host with an activated immune system. While the complex nature of *in vivo* infection means that it remains possible that there are yet other, unidentified, factors that also contribute to the differential induction of GFP fluorescence observed, the apparent specificity of the *rv2390c*'::GFP reporter supports the notion of [Cl^−^] and pH being at least two of the major drivers of the phenotype observed. This is also consistent with the increase acidification of *Mycobacterium*-containing phagosomes in activated MØs reported previously [4], [27], [30], and supports the contention that the bacteria are delivered live to a compartment that represents a more hostile environment.

**Figure 6:**
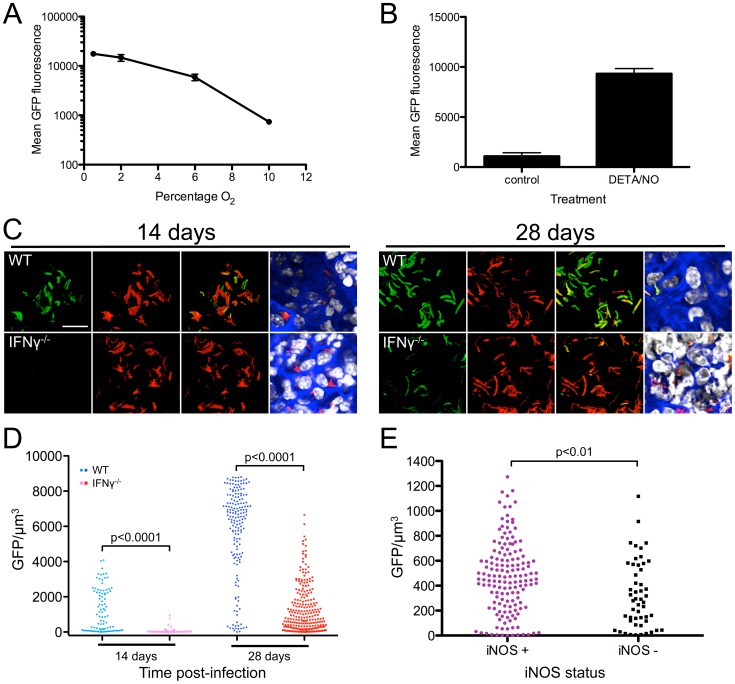
A reporter Mtb strain responsive to O_2_ tension and NO. (A) *hspX*'::GFP responds to hypoxia. Erdman(*hspX*'::GFP, *smyc*'::mCherry) was grown in 7H9 broth in stirred cultures and exposed to changing O_2_ tension (10% to 0.05%) stepwise over 6 days. Samples at indicated O_2_ tensions were fixed and GFP signal analyzed by FACS. Data are shown as means ± SD from 3 independent experiments. (B) *hspX*'::GFP responds to NO. Erdman(*hspX*'::GFP, *smyc*'::mCherry) was grown in 7H9 broth in stirred, aerated, cultures +/− 100 µM DETA/NO for 2 days. Samples were fixed and GFP signal analyzed by FACS. Data are shown as means ± SD from 3 independent experiments. (C and D) Differential induction of *hspX*'::GFP during Mtb infection of WT vs. IFNγ^−/−^ mice. WT or IFNγ^−/−^ C57BL/6J mice were infected with Erdman(*hspX*'::GFP, *smyc*'::mCherry) for up to 28 days. (C) shows 3D confocal images from a 14 or 28 day infection. All bacteria are marked in red (*smyc*'::mCherry), the reporter is shown in green (*hspX*'::GFP), nuclei are shown in grayscale (DAPI), and phalloidin staining of f-actin is shown in blue. Scale bar 10 µm. (D) shows quantification of the GFP/µm^3^ signal for each bacterium measured from multiple 3D confocal images, at 14 or 28 days post-infection. Each point on the graph represents a bacterium or a tightly clustered group of bacteria (circles – Mtb in WT mice, squares – Mtb in IFNγ^−/−^ mice). p-values were obtained with a Mann-Whitney statistical test. (E) Immune activation upregulates *hspX*'::GFP induction. C57BL6/J WT mice were infected with Erdman(*hspX*::GFP, *smyc*'::mCherry) for 28 days. Graph shows quantification of the bacterial GFP/µm^3^, determined as in (D), in Mtb present in iNOS-positive vs. negative regions. p-values were obtained with a Mann-Whitney statistical test.

In order to further validate the utility of reporter strains for studying Mtb infection, we performed additional experiments to examine the possibility of generating a second, independent reporter Mtb strain that would respond to different environmental cues from the *rv2390c*'::GFP reporter strain. In particular, we pursued *in vivo* studies with a *hspX* promoter-driven reporter strain. *hspX* is a much-studied Mtb gene often used as a marker of expression of the *dos* regulon, known to respond to hypoxia and NO [19]–[22]. As expected, *in vitro*, GFP induction of an Erdman(*hspX*'::GFP, *smyc*'::mCherry) reporter strain varied with O_2_ tension and NO (Figures 6A and 6B). Confocal microscopy analyses of lung tissue from mice infected with Erdman(*hspX*'::GFP, *smyc*'::mCherry) showed significantly greater induction of Mtb reporter GFP fluorescence in WT vs. IFNγ^−/−^ mice at both 14 and 28 days post-infection (Figures 6C and 6D). We also observed much greater induction of *hspX*'-driven GFP signal at 28 days vs. 14 days post-infection, in accord with the reported time-frame of iNOS synthesis during Mtb infection in WT mice (Figures 6C and 6D) [31]. Immunofluorescent staining of iNOS further showed significantly higher *hspX*'-driven GFP fluorescence in Mtb residing in iNOS-positive vs. negative regions in WT mice (Figure 6E). Together with the Erdman(*rv2390c*'::GFP, *smyc*'::mCherry) results above, these experiments illustrate that both reporter Mtb strains reliably detect and respond to localized regions of immune activation *in vivo*, and support the usefulness of reporter Mtb strains for studies of Mtb-host interactions.

### Concluding remarks

Our finding that Mtb can utilize Cl^−^ as an environmental cue, in synergy with pH, is a first illustration of a pathogen exploiting interlinked host signals during phagosome maturation. Importantly, Mtb responds to these cues not just *in vitro* but also during *in vivo* infection, where these signals are modulated by immune activity of the host. Most studies on Mtb and its response to environmental cues have centered on *in vitro* assays and homogeneous bacterial cultures.

While these constitute an important foundation they provide little insight into how Mtb senses and responds to environmental cues *in vivo*, where the heterogeneity linked to location and immune activation is critical in determining the productiveness of the diverse subpopulations of Mtb present in an infected host [32]. In the current study we validated the two reporter strains for their ability to respond to stresses relevant to their survival *in vivo*. Using confocal microscopy and rigorous quantification of GFP fluorescence at the level of the individual bacterium, we were able to probe infected mouse tissue and demonstrate that: (1) GFP expression level was linked to immune activation by IFNγ, (2) bacteria in regions that stained positive for the activation marker iNOS exhibited higher levels of GFP expression, and (3) the heterogeneity amongst the bacterial population was as marked as predicted [32], and can only be revealed by panels of reporter bacteria such as the ones developed in this current study. We feel that these strains represent a new generation of tools to probe the fitness of Mtb *in vivo*. These strains should enable us to functionally dissect the TB granuloma to identify privileged regions of bacteria growth, or hostile areas of immune containment. We also predict that these strains will be valuable in probing for drug action and tissue penetrance, through enhanced stress, as one tries to improve drug availability *in vivo*.

Extending beyond Mtb, our results also have potential implications for other intracellular organisms that similarly experience compartments with a range of decreased pH, such as the bacteria *Coxiella burnetti* [33] and *Brucella* [34], and the parasite *Leishmania* [35]. Might these microbes also respond to Cl^−^, and is the ability to use Cl^−^ and pH as synergistic cues a more widespread phenomenon? In bacterial studies, Cl^−^ has largely been examined only within the context of salt tolerance and osmolarity. Few reports have studied Cl^−^ itself in the context of bacterial-host interactions, although Radtke and colleagues proposed that increased [Cl^−^] aided *Listeria monocytogenes* phagosomal escape by increased activation of listeriolysin O [36].

Our study further raises the question of what roles other common ions might have on bacterial-host interactions. Although ions, such as iron, that serve as essential micronutrients and are actively sequestered by the host have long been recognized as important focal points for bacterial-host interactions [37], the possible impact of more common ions, like Cl^−^, remain largely unstudied. In addition to Cl^−^, we speculate that other common ions, such as K^+^, might also act as a signal for infecting bacteria. There are several known bacterial K^+^ transporters [38], and these also impact on important aspects such as pH and membrane potential [39]. Indeed, K^+^ transporter mutants in several bacterial species, including Mtb, have been reported to be attenuated in colonization of their host [40], [41]. We propose that further study of common ions and their possible role as environmental signals for microbes will yield many more as yet undiscovered aspects of the bacterial-host interface.

## Materials and Methods

### Ethics statement

All animal procedures were conducted in strict compliance with the National Institutes of Health “Guide for the Care and Use of Laboratory Animals”. The animal protocol was reviewed and approved (protocol number 2011-0086) by the Institutional Animal Care and Use Committee, Cornell University, under the guidelines of the Association for Assessment and Accreditation of Laboratory Animal Care, US Department of Agriculture, and the Public Health Service guidelines for the care and use of animals as attested by the National Institutes of Health. All efforts were made to minimize suffering.

### Cell culture

Bone marrow-derived MØs were isolated from C57BL/6J WT mice (Jackson Laboratories), and maintained in DMEM (Corning cellgro) containing 10% FBS (Thermo Scientific), 20% L-cell conditioned media, 2 mM L-glutamine, 1 mM sodium pyruvate and antibiotics (penicillin/streptomycin) (Corning cellgro), at 37°C in a 7% CO_2_ atmosphere. Monocytes isolated from peripheral blood mononuclear cells (Elutriation Core Facility, University of Nebraska Medical Center) were grown in DMEM containing 10% human serum (SeraCare Life Sciences), 2 mM L-glutamine, 1 mM sodium pyruvate and antibiotics, and allowed to fully differentiate into MØs before use in assays.

### Cl^−^ measurement assays

Generation of Cl^−^ and Cl^−^/pH sensor beads are described in the Supplementary Materials and Methods. For plate reader assays, 2×10^5^ MØs/well were seeded in a 96-well black plate (Corning Costar), and for confocal live-cell time-lapse microscopy assays, 4×10^5^ MØs/well were seeded in a Lab-Tek II 8-chambered coverglass (Nalge Nunc International). MØs were washed 3x with pre-warmed assay buffer (PBS, pH 7.2, 5% FBS, 5 mM dextrose, 1 mM calcium acetate, 1.35 mM K_2_SO_4_, 0.5 mM MgSO_4_), and sensor beads added at ∼2–5 beads/MØ in assay buffer. Acquisition of data on a plate reader or by confocal imaging was initiated within 2–3 minutes of bead addition. A Molecular Devices Gemini EM fluorescence plate reader was used for bottom read signal detection (BAC – Ex. 365 nm/Em. 505 nm, AF594 – Ex. 590 nm/Em. 617 nm, pHrodo – Ex. 560 nm/Em. 585 nm), with 4 replicate wells/condition, and temperature control at 37°C. In experiments to establish a calibration curve, at the end of the assay (2 hrs) described above, the MØs were washed 3x with pre-warmed Cl^−^-free buffer (1.54 mM KH_2_PO_4_, 2.71 mM Na_2_HPO_4_, 69 mM Na_2_SO_4_, 5 mM dextrose, 1 mM calcium acetate, 1.35 mM K_2_SO_4_, 0.5 mM MgSO_4_), and then placed in buffer supplemented with specific [NaCl], 200 nM bafilomycin A1 (Sigma), 10 µM nigericin (Calbiochem), and 10 µM monensin (Enzo Life Sciences). After incubation to allow equilibration, the BAC and AF594 fluorescence signals were read on a plate reader as above.

For live-cell time-lapse microscopy, cells were imaged with a Leica SP5 confocal, equipped with a stage enclosed temperature control system. A 364 nm laser line was used for excitation of BAC fluorescence, a 594 nm laser line for Alexa Fluor 594 fluorescence, and a 543 nm line for pHrodo. Emission detection was set at +/−15 nm of the peak emission λ in each case. 10 z-slices over a 12 µm range were acquired at each time point, using the Leica Application Suite Advanced Fluorescence program. Volocity software (PerkinElmer) was used for analysis and tracking of individual beads.

### Mtb strains and culture

The Mtb strain CDC1551 was the parental strain for all *in vitro* and MØ infection experiments. Strains used in mice infections were in the Erdman strain background. Routine culture of Mtb was as previously described [24]. The *phoP*::Tn mutant was from BEI (#NR-14776), and has been previously described [24]. Details of the construction of a CDC1551 Δ*phoPR* mutant and its complemented strain are described in the Supplementary Materials and Methods.

### Microarray and qRT-PCR analyses

Log-phase Mtb (OD_600_∼0.6) was used to seed 10 ml cultures at OD_600_ = 0.3 in 7H9 media buffered at pH 7.0, +/−250 mM NaCl, in standing vented T-25 flasks. RNA samples were collected after 4 hours of treatment, and five biological replicates were tested. RNA isolation, amplification, labeling and analyses by microarrays were carried out as previously described [5]. This microarray dataset is available in the ArrayExpress database under accession number E-MTAB-1374, and on the TB Database website [42]. qRT-PCR experiments were conducted on cDNA generated from amplified RNA as previously described [24].

### Fluorescent reporter Mtb strains and *in vitro* assays

To generate CDC1551(*rv2390c*'::GFP), a 704 bp region immediately upstream of *rv2390c* was PCR amplified, placed in front of GFPmut2 [43] in a modified replicating plasmid pSE100 [24], and transformed into CDC1551. The *rv2390c*'::GFP, *smyc*'::mCherry reporter strain was constructed by cloning of *rv2390c*'::GFP into the replicating plasmid pCherry3 [25], and transformation into CDC1551 or Erdman. To construct the Erdman(*hspX*'::GFP, *smyc*'::mCherry) reporter, a 558 bp region upstream of the *hspX* start codon was PCR amplified and cloned upstream of GFPmut2 in the pSE100 vector. The *hspX*'::GFP fusion was then subcloned into the pCherry3 plasmid and transformed into Erdman. Selection in all cases was carried out on 7H10 agar containing 50 µg/ml hygromycin.

For broth assays, Mtb was grown in standing vented T-25 flasks, in 10 ml 7H9 medium buffered at specified pH, with addition of NaCl or other compounds as stated for each experiment. pH 7.0 medium was buffered with 100 mM MOPS, while pH 5.5–6.5 media were buffered with 100 mM MES. Appropriate antibiotics were added as necessary. NO assays were done in stirred, aerated, cultures and used the NO donor DETA NONOate (Cayman Chemicals) at 100 µM. Hypoxia experiments were conducted in 50 ml culture volumes in 125 ml duo-capped Erlenmeyer flasks (BD Biosciences) with stirring using a magnetic stir bar. Cultures were placed in a hypoxia chamber with adjustable O_2_ and CO_2_ controls (BioSpherix), set on a magnetic stirrer within a 37°C incubator. CO_2_ was set at 7%, while O_2_ levels were adjusted as required. For all *in vitro* assays, samples were fixed with 4% paraformaldehyde and GFP fluorescence read on a BD FACS LSR II. FACS data were analyzed using FloJo (Tree Star, Inc).

### Macrophage infections

Infection of murine bone marrow-derived MØs with Mtb were carried out as previously described [24]. Where needed, MØs were activated by treatment with 100 U/ml IFNγ and 10 ng/ml LPS. For infection with CDC1551(*rv2390c*'::GFP, *smyc*'::mCherry) pre-induced with Cl^−^, the bacteria were grown in the presence of 250 mM NaCl for 6 days prior to MØ infection. Bacteria were at log-phase when MØs were infected. Samples were fixed, imaged and analyzed by confocal microscopy as described below.

### Mouse Mtb infections

All animal experiments were carried out in accordance with NIH guidelines, and with the approval of the Institutional Animal Care and Use Committee of Cornell University. C57BL/6J WT mice and their isogenic IFNγ^−/−^ derivatives (Jackson Laboratories) were infected with 10^3^ CFU of Erdman(*rv2390c*'::GFP, *smyc*'::mCherry) or Erdman(*hspX*'::GFP, *smyc*'::mCherry) via an intranasal delivery method. This was accomplished by lightly anesthetizing the mice with isoflurane and administering the bacterial inoculum in a 25 µl volume onto both nares. At sacrifice, the lungs were removed and fixed in 4% paraformaldehyde overnight.

### Confocal immunofluorescence microscopy

For MØ infections, Mtb infected cells on glass coverslips were fixed overnight at indicated time points with 4% paraformaldehyde. Nuclei were visualized with DAPI (Invitrogen). For mouse infections, whole lung lobes were fixed overnight with 4% paraformaldehyde, and stored in PBS prior to processing. Details of sample processing and antibodies used for confocal microscopy imaging are described in the Supplementary Materials and Methods. Samples were imaged with a Leica SP5 confocal microscope, and z-stacks reconstructed into 3D using Volocity software. For quantification of reporter Mtb signal, the fluorescence voxel volume of each bacterium was measured via the mCherry channel, with the corresponding sum of the GFP signal for that bacterium simultaneously measured. Settings for the GFP channel were maintained during imaging of samples within experimental sets to allow comparison of values. At least 100 bacteria were quantified for each condition. Statistical differences between data sets were determined by a non-parametric Mann-Whitney test.

## Supporting Information

Text S1 **Text S1 contains supplemental Materials and Methods, supplemental figures and legends, and the supplemental video legends.**
(PDF)

Video S1 **Time-lapse of murine bone-marrow derived MØ phagocytosis of BAC/AF594 beads.** Time-lapse movie showing phagocytosis of BAC/AF594 beads. BAC (green)/AF594 (red) beads were added to murine bone-marrow derived MØs and imaged every 2 minutes for 60 minutes. 10 z-sections were imaged at each time point, and merged. The movie is compressed into 3 seconds.
(MOV)

Video S2 **Time-lapse of BAC/AF594 beads in media alone.** Time-lapse movie of BAC/AF594 beads in media alone. BAC (green)/AF594 (red) beads were placed in assay buffer and subjected to the same number of exposures as the MØ phagocytosis experiment in Video S1. The movie is compressed into 3 seconds.
(MOV)

Video S3 **Time-lapse of murine bone-marrow derived MØ phagocytosis of BAC/pHrodo beads.** Time-lapse movie showing phagocytosis of BAC/pHrodo beads. BAC (green)/pHrodo (red) beads were added to murine bone-marrow derived MØs and imaged every 2 minutes for 60 minutes. 10 z-sections were imaged at each time point, and merged. The movie is compressed into 3 seconds.
(MOV)

Video S4 **Time-lapse of BAC/pHrodo beads in media alone.** Time-lapse movie of BAC/pHrodo beads in media alone. BAC (green)/pHrodo (red) beads were placed in assay buffer and subjected to the same number of exposures as the MØ phagocytosis experiment in Video S3. The movie is compressed into 3 seconds.
(MOV)
