# Supplementary material for: Mycobacterium tuberculosis Responds to Chloride and pH as Synergistic Cues to the Immune Status of its Host Cell
Source: PLoS Pathog. 2013 Apr 4;9(4):e1003282. doi: 10.1371/journal.ppat.1003282 (PMC3616970; doi:10.1371/journal.ppat.1003282)
Supplement: Text S1 — contains supplemental Materials and Methods, supplemental figures and legends, and the supplemental video legends. (PDF) [file ppat.1003282.s001.pdf]

SUPPLEMENTAL MATERIALS AND METHODS

Generation of Cl^- sensor beads

The fluorescent Cl^- sensitive compound 10,10'-Bis[3-carboxylpropyl]-9,9'-biacridinium (BAC) [1] was synthesized as its succinimidyl derivative as a trifluoroacetate salt (The Chemistry Research Solutions LLC). To generate Cl^- sensor beads, carboxylated, 3 µm silica beads (Kisker Biotech) were first covalently linked to human IgG (Sigma-Aldrich) and defatted BSA as previously described [2]. 0.5 mg of BAC was then added to 1 ml of the beads in coupling buffer (0.1 M sodium borate, pH 8) and incubated with agitation for 1 hr. After washing twice with coupling buffer, the BAC-labeled beads were resuspended in 1 ml of coupling buffer with 1 µg of Alexa Fluor 594-SE (Invitrogen), and incubated with agitation for a further 1 hr. Finally, the beads were washed 3x with coupling buffer, and stored in coupling buffer at 4°C. Alexa Fluor 594 is an ideal calibration fluorophore for our assays, as its fluorescence is not affected by changes in pH or [Cl^-], and its fluorescence spectra is well separated from BAC. Beads were washed and resuspended in water before use in assays. Dual Cl^- and pH sensing beads were generated similarly, with addition of 25 µg of pHrodo-SE (Invitrogen) to BAC-labeled beads.

In vitro Cl^- sensor bead assays

BAC/AF594 beads were added to buffer (1.54 mM KH_2PO_4, 2.71 mM Na_2HPO_4, 69 mM Na_2SO_4, 5 mM dextrose, 1 mM calcium acetate, 1.35 mM K_2SO_4, 0.5 mM $MgSO_4$) supplemented with specific [NaCl], allowed to equilibrate, and fluorescence signals read on a Molecular Devices Gemini EM fluorescence plate reader (BAC – Ex. 365 nm/Em. 505 nm, AF594 – Ex. 590 nm/Em. 617 nm). For *in vitro* pH tests, BAC/AF594 beads were added to water

buffered with 100 mM MES, and pH adjusted to values between 5 and 7. After equilibration, fluorescence signals were read on a plate reader as above.

rv2390c'::GFP reporter switch-off assay

CDC1551(*rv2390c*'::GFP) was grown in standing vented T-25 flasks, in 10 ml 7H9 medium buffered at pH 7 with 100 mM MOPS, 50 µg/ml hygromycin, +/- 250 mM NaCl. After 10 days, an aliquot of the bacteria were pelleted, and re-inoculated into fresh 10 ml 7H9 medium, pH 7, 50 µg/ml hygromycin, without added NaCl. Samples were removed and fixed with 4% paraformaldehyde as required over a 7-day time-course. GFP fluorescence was read on a BD FACS LSR II, and data analyzed using FloJo (Tree Star, Inc).

Construction of Mtb mutant strains

A CDC1551 Δ*phoPR* mutant was constructed by PCR amplification of ~1 kb regions flanking *phoPR*, cloning into the plasmid pYUB854 on either side of its hygromycin resistance cassette, and transformation into streptomycin-resistant *rpsL* CDC1551. Transformants were selected on 7H10 agar containing 50 µg/ml hygromycin and 100 µg/ml streptomycin, and mutants verified by sequencing of the deletion junctions. Complementation of the Δ*phoPR* mutant (*phoPR**) was accomplished by cloning the *phoPR* open reading frame and its native promoter into the integrative vector pMV306, which integrates the fragment at the *attB* site. Selection for *phoPR** transformants was on 7H10 agar containing 25 µg/ml kanamycin and 50 µg/ml hygromycin.

Sample processing for confocal immunofluorescence microscopy

Thick sections of a lung lobe (~ 0.5 mm) were cut by hand with a razor, then blocked and permeabilized by incubating for 1 hour at room temperature in PBS + 3% BSA + 0.1% Triton X-100 ("blocking buffer"). Samples were incubated with primary antibodies overnight at 4°C, washed 3x with blocking buffer, then incubated with secondary antibodies for 2 hours at room temperature. After washing 3x with blocking buffer, samples were mounted with Vectashield mounting medium (Vector Labs). Rabbit anti-iNOS (BD Transduction Labs) was used at 1:100, and Alexa fluor 514 goat anti-rabbit (Invitrogen) used at 1:200 for secondary detection. Alexa fluor 647 conjugated phalloidin (Invitrogen) was used for visualization of the actin cytoskeleton and nuclei visualized with DAPI.

SUPPLEMENTAL REFERENCES

1. Sonawane ND, Thiagarajah JR, Verkman AS (2002) Chloride concentration in endosomes measured using a ratioable fluorescent Cl^- indicator: evidence for chloride accumulation during acidification. J Biol Chem 277: 5506-5513.
2. Yates RM, Russell DG (2008) Real-time spectrofluorometric assays for the lumenal environment of the maturing phagosome. Methods Mol Biol 445: 311-325.

SUPPLEMENTAL FIGURE LEGENDS

Figure S1. BAC is a Cl^--sensitive, pH-insensitive fluorescent compound.

(A) BAC fluorescence decreases with increasing [Cl^-]. IgG beads coated with BAC were placed in buffer containing specific [NaCl], and fluorescence measured on a microplate reader. Data are presented as F_0/F, where F_0 is the BAC fluorescence at [NaCl] = 0 mM, and F is the fluorescence at a given [NaCl]. Data are shown as means ± SD from 4 wells.

(B) BAC fluorescence is unaffected by changes in pH. IgG beads coated with BAC were placed in water buffered with 100 mM MES and adjusted to particular pH values with NaOH.

Fluorescence was measured on a microplate reader, and data are presented as F_0/F, where F_0 is the BAC fluorescence at pH 7.0, and F is the fluorescence at a given pH. Data are shown as means ± SD from 4 wells.

Figure S2. Calibration of AF594/BAC fluorescence with [Cl^-]

Murine bone marrow-derived MØs (A) or MØs derived from human monocytes (B) were incubated with AF594/BAC beads for 2 hours, before exposure to buffers containing specific [NaCl] and a bafilomycin/ionophore mixture (see Experimental Methods), equilibration, and fluorescence signal reading on a plate reader. Data are shown as means ± SD from 4 wells. A polynomial regression curve was fitted to the data. $R^2 = 0.98$ (A) and $R^2 = 0.99$ (B).

Figure S3. Phagosomal Cl^- accumulation requires a [H^+] gradient

(A) Inhibition of phagosomal acidification prevents Cl^- accumulation. BAC/AF594 beads were added to murine bone marrow-derived MØs either in the absence ("control") or presence ("+BafA1") of 200 nM bafilomycin A1. BAC (Cl^--sensitive) and AF594 (calibration fluorophore) fluorescence were tracked with a microplate reader over time. Data are shown as means ± SD from 6 wells.

(B) Cl^- accumulation is reversed by abrogation of phagosomal [H^+] gradient. BAC/AF594 beads were added to murine bone marrow-derived MØs in the absence of bafilomycin A1. After 120 minutes (vertical dashed line), 200 nM bafilomycin A1 was added to half the samples ("switch BafA1"). The other half of the samples received fresh media without bafilomycin A1 ("control"). BAC and AF594 fluorescence were tracked with a microplate reader over time. Data are shown as means ± SD from 6 wells.

Figure S4. Overlap in subset of Mtb genes upregulated during Cl^- and acid exposure

qRT-PCR of gene expression in WT grown in media at pH 7.0 + 250 mM NaCl, or pH 5.7, as compared to WT grown in media at pH 7.0, for 4 hrs. Data are shown as means ± SD from 3 technical replicates.

Figure S5. Specificity of *rv2390c*'::GFP induction

CDC1551(*rv2390c*'::GFP) was grown *in vitro* in media at pH 7.0 (control, circles), supplemented with 250 mM NaCl (squares), 250 mM KCl (triangles), 250 mM arginine-HCl (inverted triangles), 167 mM Na_2SO_4 (diamonds), or 300 mM sucrose (open circles). Samples were taken over time, fixed, and GFP signal analyzed by FACS. Data are shown as means ± SD from 3 independent experiments.

Figure S6. Induction of *rv2390c*'::GFP fluorescence is reversible

CDC1551(*rv2390c*'::GFP) was first grown *in vitro* in media at pH 7.0 ("control"), or in media at pH 7.0 + 250 mM NaCl for 10 days. Aliquots were then taken and re-inoculated into fresh media at pH 7.0 without added NaCl (day 0 post-media switch) for all samples. Samples were taken over time, fixed, and GFP signal analyzed by FACS. Data are shown as means ± SD from 3 independent experiments

Figure S7. *rv2390c*'::GFP is not induced by NO or hypoxia

(A) *rv2390c*'::GFP is not induced by NO. CDC1551(*rv2390c*'::GFP) was grown *in vitro* in 7H9 broth at pH 7.0, in stirred, aerated, cultures +/- 100 µM DETA/NO for 2 days. Samples were

fixed and GFP signal analyzed by FACS. Reporter Mtb grown in the presence of 250 mM NaCl ("NaCl") is shown as a positive control for GFP induction. Data are shown as means ± SD from 3 independent experiments.

(B) *rv2390c*'::GFP is not induced by hypoxia. CDC1551(*rv2390c*'::GFP) was grown *in vitro* in 7H9 broth at pH 7.0, in stirred cultures at atmospheric O_2 ("aerated"), or at 1% O_2 tension ("1% O_2") for 6 days. Samples were fixed and GFP signal analyzed by FACS. Reporter Mtb grown in the presence of 250 mM NaCl ("NaCl") is shown as a positive control for GFP induction. Data are shown as means ± SD from 3 independent experiments.

Figure S8. Expression of *smyc*'::mCherry allows visualization of all bacteria

CDC1551(*rv2390c*'::GFP, *smyc*'::mCherry) was grown *in vitro* in media at pH 7.0 ± 250 mM NaCl for 6 days, fixed, and examined by confocal microscopy. 3D confocal images are shown, with all bacteria marked in red (*smyc*'::mCherry), and the reporter shown in green (*rv2390c*'::GFP). Scale bar 5 µm.

Figure S9. Erdman(*rv2390c*'::GFP) functions as a Cl^- and pH responsive reporter Mtb

Erdman(*rv2390c*'::GFP) was grown *in vitro* in media at pH 7.0 (control, circles), pH 7.0 + 250 mM NaCl (squares), pH 5.7 (triangles), or pH 5.7 + 250 mM NaCl (diamonds). Samples were taken over time, fixed, and GFP signal analyzed by FACS. Data are shown as means ± SD from 3 independent experiments.

SUPPLEMENTAL VIDEO LEGENDS

Video S1. Time-lapse of murine bone marrow-derived MØ phagocytosis of BAC/AF594 beads.

Time-lapse movie showing phagocytosis of BAC/AF594 beads. BAC (green)/AF594 (red) beads were added to murine bone marrow-derived MØs and imaged every 2 minutes for 60 minutes. 10 z-sections were imaged at each time point, and merged. The movie is compressed into 3 seconds.

Video S2. Time-lapse of BAC/AF594 beads in media alone.

Time-lapse movie of BAC/AF594 beads in media alone. BAC (green)/AF594 (red) beads were placed in assay buffer and subjected to the same number of exposures as the MØ phagocytosis experiment in Movie S1. The movie is compressed into 3 seconds.

Video S3. Time-lapse of murine bone marrow-derived MØ phagocytosis of BAC/pHrodo beads.

Time-lapse movie showing phagocytosis of BAC/pHrodo beads. BAC (green)/pHrodo (red) beads were added to murine bone marrow-derived MØs and imaged every 2 minutes for 60 minutes. 10 z-sections were imaged at each time point, and merged. The movie is compressed into 3 seconds.

Video S4. Time-lapse of BAC/pHrodo beads in media alone.

Time-lapse movie of BAC/pHrodo beads in media alone. BAC (green)/pHrodo (red) beads were placed in assay buffer and subjected to the same number of exposures as the MØ phagocytosis experiment in Movie S3. The movie is compressed into 3 seconds.

Fig S1.

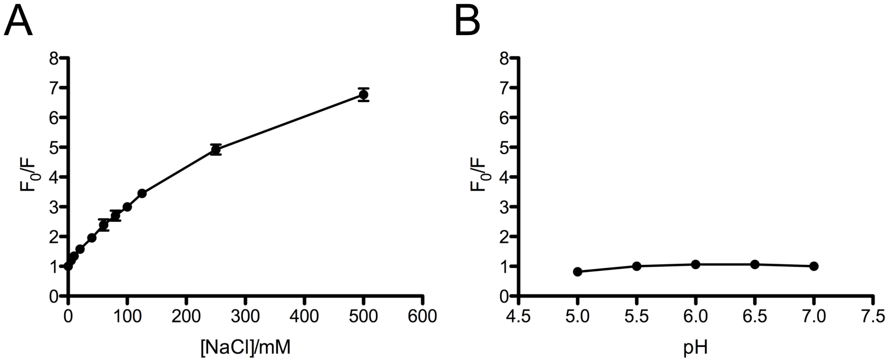

Fig S2.

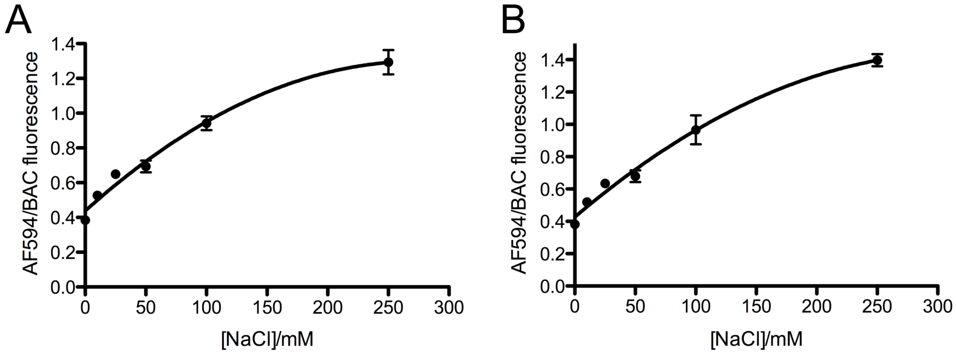

Fig S3.

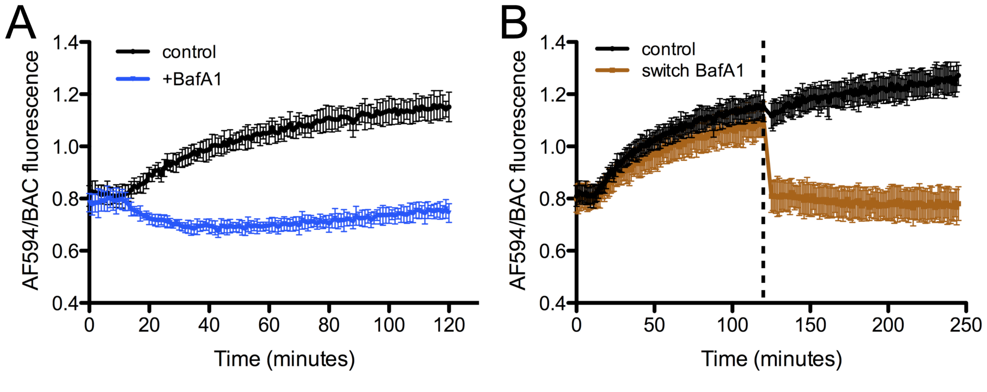

Fig S4.

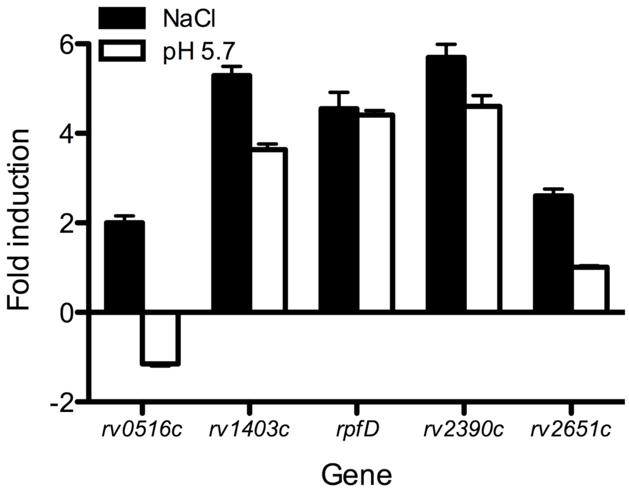

NaCl
pH 5.7
Fold induction
6
4
2
0
-2
rv0516c
rv1403c
rpfD
rv2390c
rv2651c
Gene

Fig S5.

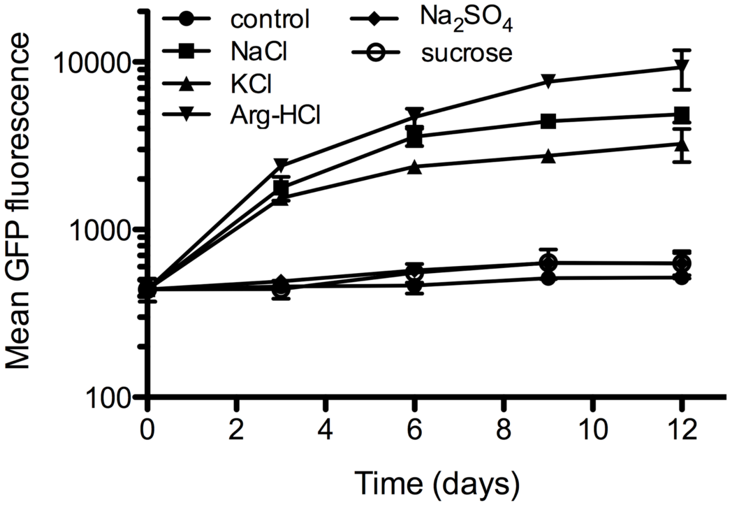

control
NaCl
KCl
Arg-HCl
Na_2SO_4
sucrose
Mean GFP fluorescence
10000
1000
100
0
2
4
6
8
10
12
Time (days)

Fig S6.

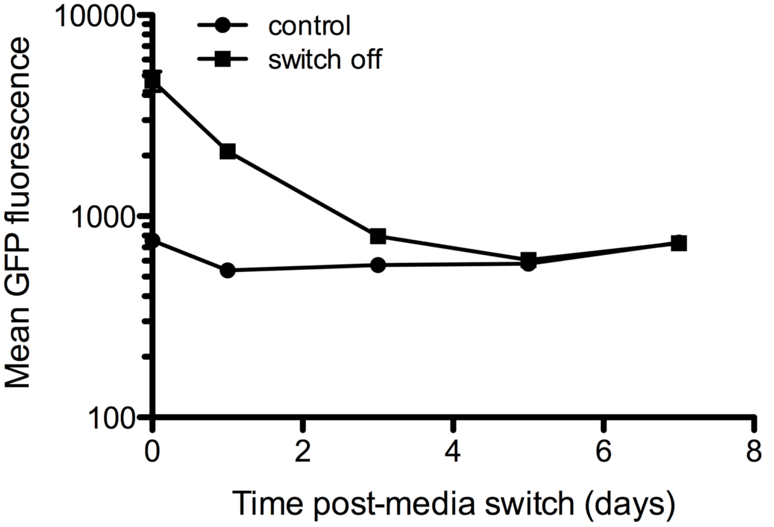
10000
1000
100
Mean GFP fluorescence
control
switch off
0
2
4
6
8
Time post-media switch (days)

Fig S7.

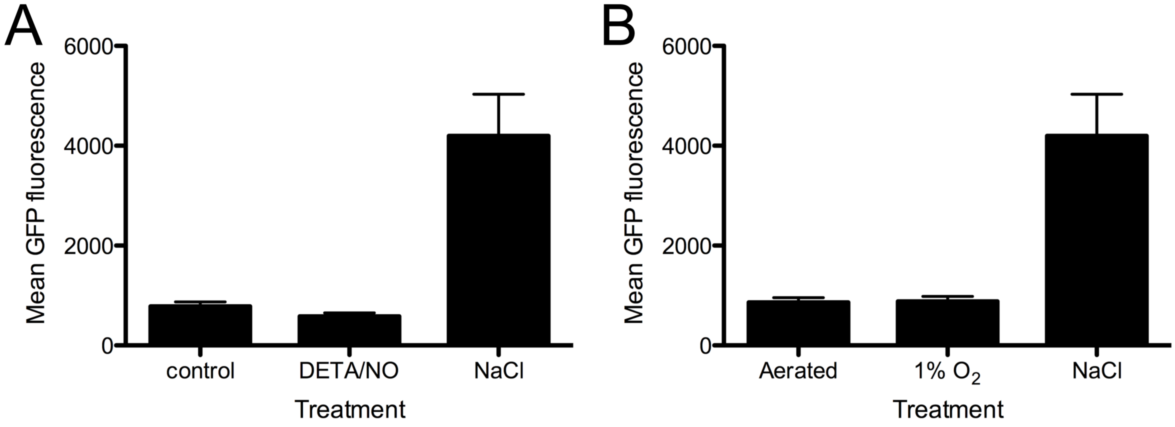
A
6000
4000
2000
0
Mean GFP fluorescence
control
DETA/NO
NaCl
Treatment
B
6000
4000
2000
0
Mean GFP fluorescence
Aerated
1% O2
NaCl
Treatment

Fig S8.

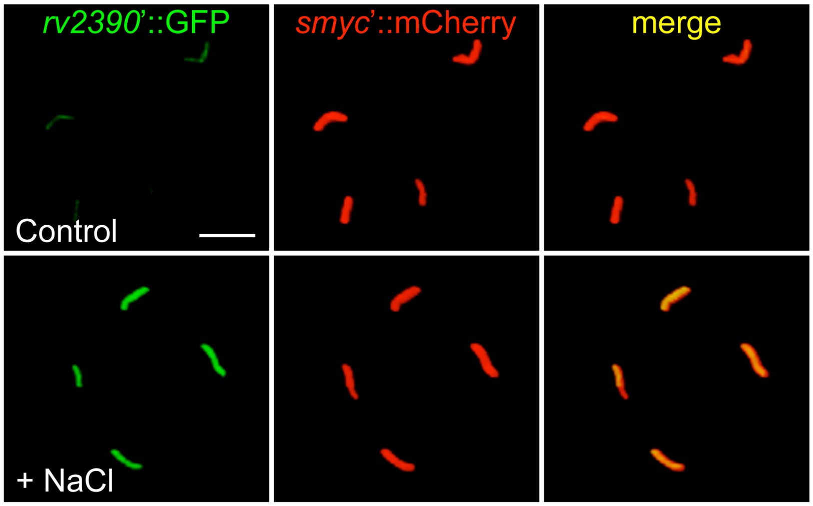
rv2390'::GFP
smyc'::mCherry
merge
Control
+ NaCl

Fig S9.

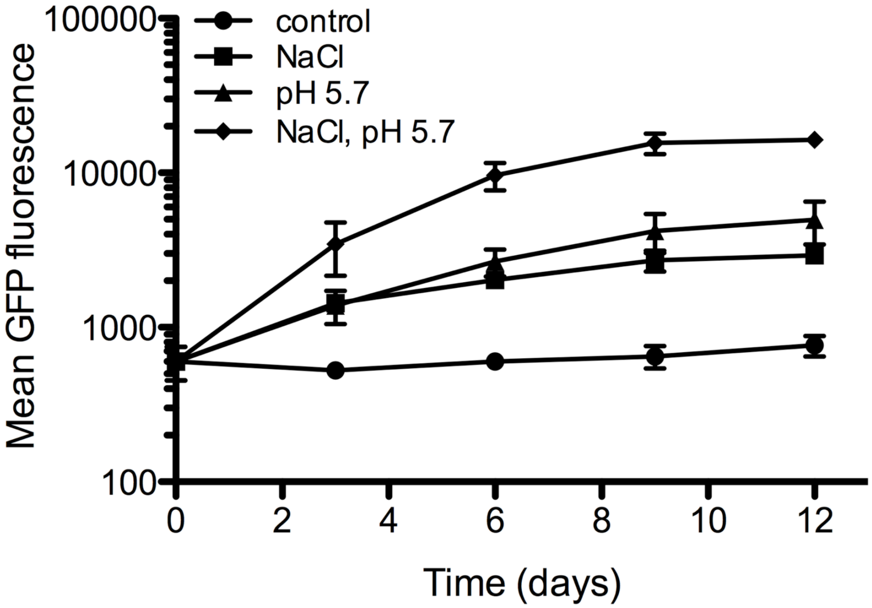
control
NaCl
pH 5.7
NaCl, pH 5.7
Mean GFP fluorescence
100000
10000
1000
100
0
2
4
6
8
10
12
Time (days)
